# Using Consolidated Framework for Implementation Research to investigate facilitators and barriers of implementing alcohol screening and brief intervention among primary care health professionals: a systematic review

**DOI:** 10.1186/s13012-021-01170-8

**Published:** 2021-11-20

**Authors:** Paul Shing-fong Chan, Yuan Fang, Martin Chi-sang Wong, Junjie Huang, Zixin Wang, Eng Kiong Yeoh

**Affiliations:** 1 JC School of Public Health and Primary Care, Faculty of Medicine, The Chinese University of Hong Kong, Hong Kong, China; 2 Department of Early Childhood Education, The Education University of Hong Kong, Hong Kong, China; 3 Center for Health Systems and Policy Research, JC School of Public Health and Primary Care, Faculty of Medicine, The Chinese University of Hong Kong, Hong Kong, China; 4 Room 508, School of Public Health, Prince of Wales Hospital, Shatin, N.T., Hong Kong, China

**Keywords:** Consolidated Framework for Implementation Research, Alcohol screening, Brief intervention, Facilitators, Barriers, Primary care, Health professionals

## Abstract

**Background:**

Alcohol screening and brief intervention (SBI) is recommended to be implemented in primary care settings to intervene against hazardous/harmful drinking. However, studies showed that the uptake rate was low in many regions/countries. This systematic review presented current findings on the facilitators and barriers of SBI implemented by health professionals in primary care settings using the Consolidated Framework for Implementation Research (CFIR).

**Methods:**

We included qualitative, quantitative, and mixed-method studies identified through four electronic databases (PubMed, MEDLINE, PsycInfo, and Web of Science) from inception to June 2020. Included articles had to address barriers and facilitators of SBI implementation and provide sufficient details that the CFIR domains could be identified and data were abstracted using a standardized extraction form.

**Results:**

A total of 74 studies published from 1985 to 2019 were finally analysed and summarized. The most common facilitators were knowledge and positive beliefs about SBI (characteristics of the individuals) and available resources (inner setting). In contrast, the most common barriers were cost related to implementing SBI (intervention characteristics), negative beliefs about SBI (characteristics of the individuals), and lack of self-efficacy in implementing SBI (characteristics of the individuals). It could be observed that factors related to the inner setting and characteristics of individuals were extensively studied whilst the process received the least attention.

**Conclusions:**

Most of the facilitators and barriers are modifiable. Additionally, most literature focused on various kinds of available assets to implement SBI. To promote the spread of SBI implementation, more high-quality studies on the implementation process are needed. This systematic review could serve as a reference framework for health authorities to devise strategies for improving the implementation of SBI in primary care settings.

**Trial registration:**

This systematic review was registered in PROSPERO (CRD42021258833).

**Supplementary Information:**

The online version contains supplementary material available at 10.1186/s13012-021-01170-8.

Contributions to the literature

- There is a lack of using implementation frameworks to investigate facilitators or barriers to implementing SBI in previous studies. The Consolidated Framework for Implementation Research was used as a systematic tool to analyse and summarize the data in this review.
- This review provides the most up-to-date synthesis of findings of existing studies about facilitators or barriers of SBI implemented by health professionals in primary care settings.
- This systematic review serves as a reference framework for health authorities to devise strategies for improving the implementation of SBI in primary care settings.

## Introduction

Harmful use of alcohol is a known risk factor for more than 200 types of diseases and injuries [1]. In light of this, alcohol screening and brief intervention (SBI) techniques have been developed in a bid to rectify the situation that excessive alcohol use causes harm. SBI measures a person’s level of alcohol consumption and provides brief interventions based on their drinking level [2].

The World Health Organization (WHO) stressed the need to increase coverage of SBI in order to enable early identification and intervention against hazardous/harmful drinking behaviour before serious consequences happen [3]. Across countries, primary care professionals are in a unique and privileged position to identify and intervene against hazardous/harmful drinking [4, 5]. A meta-analysis of 34 randomized controlled trials (RCTs) showed that stand-alone SBI in primary care settings had a significant and moderate effect in reducing alcohol consumption among hazardous/harmful drinkers, as compared to no or minimum intervention [6]. Early identification and secondary prevention of alcohol use disorder using SBI in primary care settings are strongly recommended by the WHO [2] and other national health authorities [7–9].

However, there was a significant gap between the actual implementation and what is recommended for SBI [10]. Screening rates in European countries were low [11] and less than half of individuals engaged in hazardous drinking were identified by their general practitioners (GPs) [12]. In view of this low uptake of SBI, a number of studies have identified facilitators or barriers to practising SBI in primary care settings. The enabling factors include training [13, 14], proven efficacy of SBI [15, 16], financial incentives [17, 18], and support from government policy [15, 19]. On the other hand, a broad variety of barriers were reported, such as lack of time [20, 21], lack of counselling skills [16, 22], low availability of screening or intervention tools [23, 24], and fear of harming their relationship with the patient [25, 26]. Nevertheless, the previous studies did not use an implementation science lens to look at facilitators and barriers to implementing SBI, except for one study. That study used Greenhalgh’s conceptual framework for dissemination of innovations to explore facilitators and barriers of SBI implemented by different health professionals in the USA [27].

To our knowledge, two systematic reviews published in 2011 and 2017 summarized the facilitators and barriers to implementing SBI in various settings (e.g. trauma centres, in-patient settings, primary care settings, etc.) [28, 29]. They did not give a separate discussion on primary care settings where SBI was suggested to be implemented by the WHO. The number of studies conducted in primary care settings was relatively small in the two reviews (*n* = 31, *n* = 14). One of them only included qualitative studies. Furthermore, both systematic reviews did not use the lens of implementation science to synthesize or discuss the findings. The present systematic review included quantitative, qualitative, and mixed-method studies which focused exclusively on primary care settings. It also used an implementation science framework to synthesize the findings.

Due to the lack of application of implementation science framework in this research area, in the present systematic review, the Consolidated Framework for Implementation Research (CFIR) was used to analyse and summarize the facilitators and barriers to implementing SBI in primary care settings. The CFIR has been used to guide the systematic assessment of multi-level implementation contexts to identify facilitators and barriers that might influence implementation [30, 31]. It provides a comprehensive and standardized list of implementation-related constructs that can be applied across the spectrum of implementation research [30, 31]. The CFIR consists of five domains, including intervention characteristics (features of an intervention that might influence implementation), inner setting (features of the organization that might influence implementation), outer setting (features of external context or environment that might influence implementation), characteristics of individuals (individuals involved in implementation that might influence implementation), and implementation process (refers to the plan of implementing a given innovation, to the contents and quality of the plan and how it has been adhered to during the actual implementation process). Making use of the CFIR is helpful to generalize the findings across contexts [30].

The present systematic review included quantitative, qualitative, and mixed-method studies which focused exclusively on facilitators and barriers to implementing SBI in primary care settings. It also used the implementation science framework, the CFIR, to synthesize the findings in a systematic manner, which could serve as a reference framework for health authorities to devise strategies to improve the implementation of SBI in primary care settings.

## Methods

This systematic review was conducted according to a pre-registered protocol in PROSPERO (CRD42021258833) and the PRISMA guideline [32].

### Search strategy

Articles were identified by searching the electronic databases PubMed, MEDLINE, PsycInfo, and Web of Science, covering the publication periods from inception to June 2020. Implementing SBI in primary care settings started in the 1980s as a result of the WHO international collaborative project on SBI [33]. The guideline/recommendation and practice of SBI in primary care settings have not changed significantly since then. The Boolean operator was used in the search strategy conducted, using “OR” and/or “AND” to link search terms. The asterisk “*” was used as a wildcard symbol appended at the end of the terms to search for variations of those terms (Additional file 1).

### Selection criteria

The articles included in this review were original, quantitative, qualitative, or mixed-method studies published in peer-reviewed journals. The studies examined the facilitators and/or barriers of alcohol screening or alcohol brief intervention implemented by healthcare professionals (physicians, nurses, and other health workers) in primary care settings. In this review, barriers refer to obstacles that hinder health professionals from performing SBI and facilitators refer to enabling factors for health professionals to perform SBI. We excluded articles that focused on: (1) efficacy of alcohol screening and brief intervention; and (2) alcohol screening combined with other drugs’ screening.

### Quality assessment and analysis

The information from the included articles was assessed by the Mixed-Method Appraisal Tool (MMAT) version 2011 with detailed descriptions of the rating [34]. A data extraction form was used which included reference ID, first author, publication year, title, country, study design, participants, sample size, facilitators, barriers, CFIR domains, and constructs. We used a thematic approach that the facilitators and barriers were coded on the CFIR framework. A six-step data synthesis process of the facilitators and barriers was developed: (1) Two reviewers extracted facilitators and barriers from each article independently; (2) After extraction, they discussed to achieve a consensus on the facilitators and barriers identified in each article. In some cases, wordings of the same facilitator or barrier were slightly different in different studies. The wordings were revised and the same description for the same facilitator or barrier was used after discussion by the two reviewers; (3) Each facilitator or barrier was coded under the domains/constructs of the CFIR by the two reviewers independently; (4) After finishing the coding independently, they discussed the results, and any discrepancies were resolved through discussion; (5) The revised coding results were read and checked by the two reviewers independently to ensure all facilitators and barriers were mapped to the CFIR constructs correctly; (6) All information in the codebook was adapted to make Tables 1 and 2.

## Results

### Identification of studies

**Figure 1:**
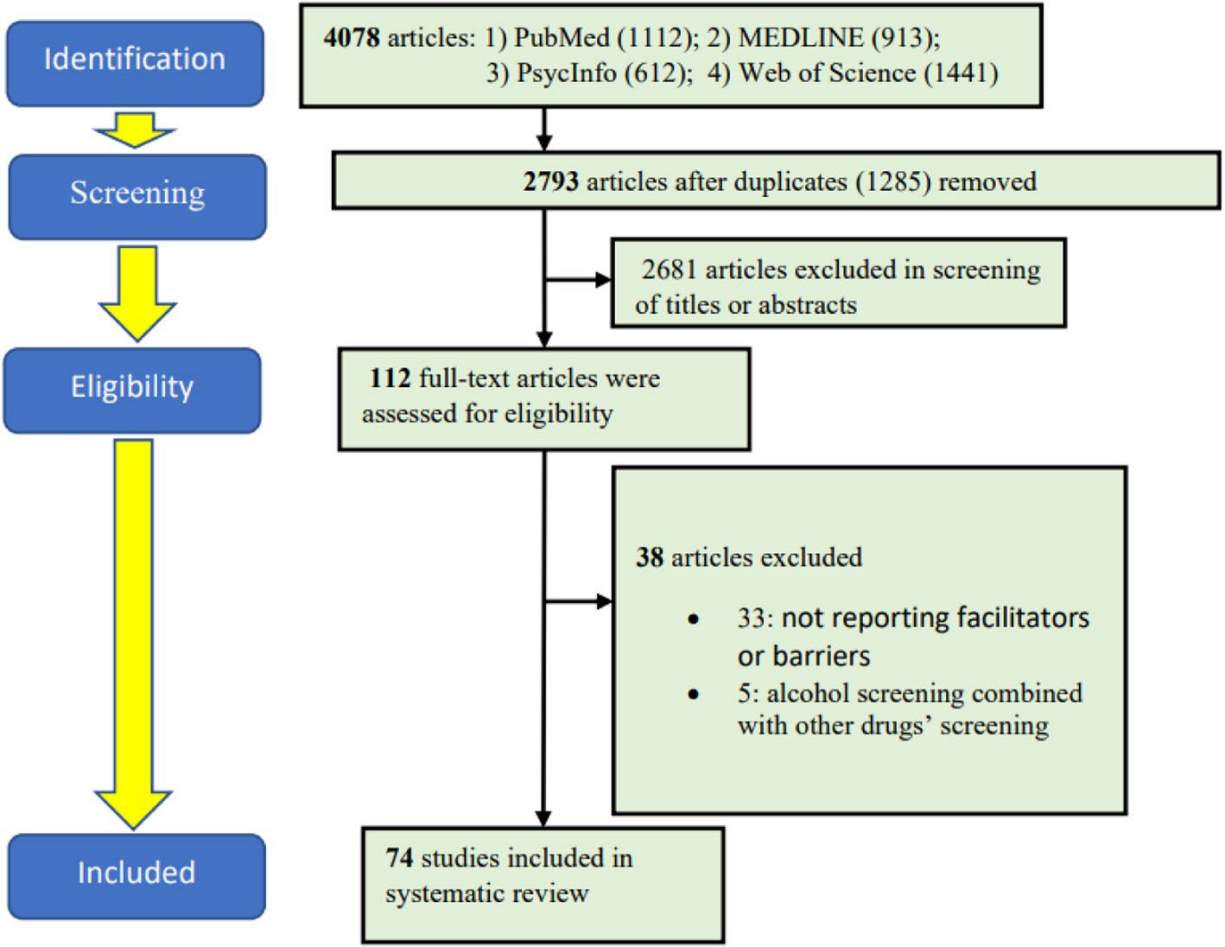
The PRISMA flowchart of the selected studies

The initial search returned 4078 citations, of which 1285 were excluded due to duplicates (Fig. 1). After that, we further removed 2681 articles after screening for titles and abstracts. We performed full-text screening on 112 articles, of which 38 articles were excluded because they did not meet the selection criteria. Figure 1 presents the PRISMA flow chart of the selected studies.

### Overview of included studies

A total of 74 studies published from 1985 to 2019 were finally analysed and summarized (Table 1) [13–26, 35–93]. Most of the studies were performed in Europe (*n* = 45, 61%), followed by North America (*n* = 14, 19%), the Asia-Pacific region (*n* = 7, 9%), South Africa (*n* = 3, 4%), South America (*n* = 1, 1%), and different continents (*n* = 4, 5%). These studies consist of 49 quantitative studies, 22 qualitative studies, and three mixed-method studies. Among these studies, 51 included physicians only (total sample size: 23597), 5 included nurses only (total sample size: 279), 9 included both physicians and nurses (3918 physicians and 3564 nurses), and 9 included different health professionals (total sample size: 3694). Regarding publication years, 10 studies were published before 2000, 39 were between 2000 and 2009, and 25 were after 2009. The overall quality of the included studies was generally good, meaning that the studies satisfied most of the criteria (Additional file 2).

**Table 1:** Characteristics, facilitators, and barriers to implementing SBI of included studies

Study (first author) and country	Study method and population	Facilitators coded by the CFIR	Barriers coded by the CFIR
Aalto (2001) [13] Finland	Survey, 84 physicians, 167 nurses	**Outer setting** *Patient needs and resources:* patient’s willingness to be asked about alcohol consumption **Inner setting** *Available resources:* practical training; lectures *Access to knowledge and information:* information about brief intervention studies **Characteristics of individuals** *Knowledge and beliefs:* positive attitudes towards discussing alcohol with patients; belief that it was worth asking about patients’ alcohol consumption; belief that detection and treatment of early phase alcohol use disorder was appropriate for their work *Self-efficacy:* self-efficacy	**Inner setting** *Available resources:* lack of training **Characteristics of individuals** *Knowledge and beliefs:* lack of knowledge in screening tools; lack of knowledge about the definition of heavy drinking; lack of knowledge about brief intervention
Aalto (2003) [35] Finland	Survey, 64 physicians	**Inner setting** *Available resources:* brief intervention tools were available **Characteristics of individuals** *Knowledge and beliefs:* belief that having the responsibility to ask about patient’s alcohol consumption *Other personal attributes:* male GPs	N.A.
Aalto (2003) a[20] Finland	Focus groups, 18 physicians, 19 nurses	N.A.	**Intervention characteristics** *Cost:* lack of time for carrying out brief intervention **Inner setting** *Access to knowledge and information:* lack of guidelines for brief intervention **Characteristics of individuals** *Knowledge and beliefs:* confusion regarding the definition of or difficulty in identifying early-phase heavy drinking; uncertainty about the justification for initiating discussion on alcohol issues with patients *Self-efficacy:* lack of self-efficacy
Aalto (2006) [36] Finland	Survey, 1909 physicians	**Characteristics of individuals** *Other personal attributes:* having a specialist licence in general practice or occupational health care	N.A.
Aira (2003) [21] Finland	Semi-structured interviews, 35 physicians	**Outer setting** *Patient needs and resources:* clear reason for consultation of the patient; patients’ specific characteristics, such as shabbiness	**Intervention characteristics** *Evidence strength and quality:* doubt about the effectiveness of intervention *Cost:* lack of time **Inner setting** *Available resources:* low availability of intervention tools **Characteristics of individuals** *Knowledge and beliefs:* fear of harming patient-physician relationship: low awareness of patients’ alcohol problems
Aira (2004) [23] Finland	Semi-structured interviews, 35 physicians	N.A.	**Intervention characteristics** *Evidence strength and quality:* doubt about the effectiveness of counselling **Outer setting** *Patient needs and resources:* patient’s denial **Inner setting** *Available resources:* lack of intervention tools **Characteristics of individuals** *Knowledge and beliefs:* belief that alcohol was not an important risk factor; belief that moderate use of alcohol was acceptable
Akvardar (2010) [37] Turkey	Survey, 135 physicians	N.A.	**Intervention characteristics** *Cost:* lack of time **Outer setting** *Patient needs and resources:* patients’ unwillingness to take advice **Characteristics of individuals** *Knowledge and beliefs:* lack of knowledge about screening tools; lack of knowledge in identifying problem drinkers; belief that alcohol issue was not an important issue in general practice; fear of harming patient-physician relationship
Amaral (2010) [38] Brazil	Focus groups, 79 different health professionals (e.g. physicians, social workers, psychologists)	**Intervention characteristics** *Design quality and packaging:* simplicity of SBI technique **Inner setting** *Structural characteristics:* the collaborative way the project was planned (teamwork)	**Intervention characteristics** *Cost:* lack of time **Outer setting** *Cosmopolitanism:* difficulty in patients’ referral **Inner setting** *Culture:* organizational culture about alcohol use *Relative priority:* competing priorities *Leadership engagement:* the variability of the institutional support due to changes in leadership **Characteristics of individuals** *Self-efficacy:* discomfort in dealing with alcohol issues
Anderson (1985) [39] UK	Survey, 312 physicians	**Characteristics of individuals** *Knowledge and beliefs:* belief that they had the right to ask patients about their alcohol drinking	**Inner setting** *Available resources:* lack of training **Characteristics of individuals** *Knowledge and beliefs:* belief that it was unrewarding *Other personal attributes:* lack of motivation
Anderson (2003) [40] Australia, Belgium, Canada, the UK, France, Italy, New Zealand, Norway, and Portugal	Survey, 1300 physicians	**Inner setting** *Access to knowledge and information:* support calls responding to questions or problems that arose during SBI implementation **Characteristics of individuals** *Knowledge and beliefs:* higher education level on alcohol; greater therapeutic commitment to working with alcohol problems *Self-efficacy:* higher role security (e.g. confidence in giving advice) in working with alcohol problems	N.A.
Anderson (2004) [14] Australia, Belgium, Canada, the UK	RCT, 632 physicians	**Inner setting** *Available resources:* training *Access to knowledge and information:* support calls responding to questions or problems that arose during SBI implementation **Characteristics of individuals** *Knowledge and beliefs:* therapeutic commitment to work with problem drinkers *Self-efficacy:* role security (e.g. confidence in giving advice appropriately)	N.A.
Anderson (2014) [17] Catalonia, Czech Republic, Italy, Netherlands, Poland, Portugal, Slovenia, and the UK	Survey, 2345 physicians	**Inner setting** *Organizational incentives and rewards****:*** financial incentives *Available resources:* screening and advice materials were available; training; **Characteristics of individuals** *Knowledge and beliefs:* higher levels of education for alcohol problems *Self-efficacy:* higher role security in working with alcohol problems (e.g. confidence in giving advice appropriately)	**Characteristics of individuals** *Knowledge and beliefs:* having their own disease model rather than prevention model of alcohol problems; belief that drinking was a personal rather than a medical responsibility
Arborelius (1995) [25] Sweden	Semi-structured interview, 13 physicians	**Inner setting** *Available resources:* concrete SBI materials	**Intervention characteristics** *Cost:* lack of time **Characteristics of individuals** *Knowledge and beliefs:* fear of harming the patient-physician relationship
Barry (2004) [41] USA	Survey, 41 physician managers	N.A.	**Intervention characteristics** *Cost:* lack of time for screening and brief intervention **Outer setting** *Patient needs and resources:* patient defensiveness for screening and brief intervention **Inner setting** *Structural characteristics:* lack of specialty staff to conduct brief intervention **Characteristics of individuals** *Knowledge and beliefs:* lack of knowledge and skills to conduct brief intervention
Beich (2002) [42] Denmark	Focus groups, 24 physicians	N.A.	**Intervention characteristics** *Cost:* considerable resources needed **Inner setting** *Compatibility:* interruptions of the natural course of consultations **Characteristics of individuals** *Knowledge and beliefs:* questioned the rationale of screening in young drinkers who may grow out of excessive drinking behaviour; *Self-efficacy:* lack of confidence in their ability to counsel patients effectively on lifestyle issues; difficulty in establishing rapport with patients
Bendtsen (2015) [43] Netherlands, Poland, Spain, Sweden, the UK	Survey, 746 different health professionals (e.g. nurses, and other staff)	**Characteristics of individuals** *Self-efficacy:* role security (e.g. confidence in giving advice appropriately)	N.A.
Berner (2007) [44] Germany	Survey, 58 physicians	**Characteristics of individuals** *Other personal attributes:* male patients; female physicians	N.A.
Brennan (2013) [45] Australia	Survey, 15 physicians	**Inner setting** *Available resources:* training **Characteristics of individuals** *Knowledge and beliefs:* knowledge of SBI *Self-efficacy:* self-efficacy	N.A.
Clement (1986) [46] UK	Survey, 71 physicians	**Outer setting** *Cosmopolitanism:* easy to refer **Characteristics of individuals** *Knowledge and beliefs:* knowledge of alcohol; belief that having the right to ask patients about their drinking *Other personal attributes:* physician’s interest in alcohol issues	**Characteristics of individuals** *Knowledge and beliefs:* belief that drinking problems would persist for a long time; belief that it was not rewarding to work with drinkers *Self-efficacy:* lack of self-efficacy in counselling patients
Clifford (2011) [47] Australia	Survey, 47 different health professionals (e.g. allied health workers, administrative staff, physicians)	**Outer setting** *Patient needs and resources:* patient’s willingness for screening **Inner setting** *Available resources:* training *Access to knowledge and information:* clear guidelines	**Outer setting** *Patient needs and resources:* patient’s resistance to referral
Coloma-Carmona (2017) [48] Spain	Survey, 60 physicians, 47 nurses	N.A.	**Intervention characteristics** *Cost:* lack of time **Outer setting** *Patient needs and resources:* patients’ dishonesty of alcohol consumption; patients’ neglect of negative consequences of alcohol consumption; patients’ unwillingness to participate in brief intervention; patients’ denial of alcohol use disorder **Inner setting** *Available resources:* lack of training
Costa (2019) [49] France	Survey, 101 physicians	**Outer setting** *Cosmopolitanism:* available referral services from community alcohol teams **Inner setting** *Available resources:* training **Characteristics of individuals** *Knowledge and beliefs:* physicians’ acceptance of controlled drinking as a therapeutic goal of treating alcohol use disorder	N.A.
Deehan (1997) [50] UK	Survey, 81 physicians	**Characteristics of individuals** *Self-efficacy:* self-efficacy	**Intervention characteristics** *Cost:* workload or lack of time; causing management problems **Characteristics of individuals** *Knowledge and beliefs:* belief that it was not a rewarding task
Deehan (1998) [51] UK	Survey, 2377 physicians	**Outer setting** *Patient needs and resources:* self-motivation of patient **Inner setting** *Available resources:* adequate training in detection of alcohol misue **Characteristics of individuals** *Knowledge and beliefs:* belief that general practice is an appropriate setting	**Intervention characteristics** *Cost:* workload or lack of time; alcohol misusers presented major management problems **Inner setting** *Structural characteristics:* lack of specialist support (teamwork) *Available resources:* lack of training in the treatment of alcohol misuse **Characteristics of individuals** *Knowledge and beliefs:* belief that alcohol misusers were unrewarding to treat *Self-efficacy:* not confident in the ability to treat alcohol misusers
Deehan (1999) [52] UK	Survey, 264 physicians, 196 nurses	**Outer setting** *Cosmopolitanism:* referral services **Inner setting** *Available resources:* training **Characteristics of individuals** *Knowledge and beliefs:* belief that general practice was an appropriate setting *Self-efficacy:* self-efficacy in working with alcohol misusers	**Intervention characteristics** *Cost:* workload or lack of time; causing management problems **Inner setting** *Available resources:* lack of training **Characteristics of individuals** *Knowledge and beliefs:* belief that it was not a rewarding task in physicians *Self-efficacy:* lack of self-efficacy in working with alcohol misusers
Farmer (2001) [53] UK	Semi-structured interview, 50 physicians	**Inner setting** *Available resources:* training **Characteristics of individuals** *Knowledge and beliefs:* knowledge of alcohol services; belief that general practice was an appropriate place to treat problem drinkers; belief that having the right to ask about patient’s drinking	**Intervention characteristics** *Cost:* lack of time **Characteristics of individuals** *Knowledge and beliefs:* belief that alcohol misuse was a social rather than health problem; pessimistic attitudes of physicians towards alcohol patients; unwilling to work with problem drinkers
Ferguson (2003) [54] USA	Survey, 42 physicians	N.A.	**Intervention characteristics** *Cost:* lack of time **Outer setting** *Patient needs and resources:* patients’ denial; lack of motivation to change *Cosmopolitanism:* lack of referral services; lack of community resources **Inner setting** *Available resources:* lack of training
Friedmann (2000) [55] USA	Survey, 853 physicians	**Characteristics of individuals** *Knowledge* *and* *beliefs:* familiarity with expert guidelines *Self-efficacy:* greater confidence in alcohol history taking *Other personal attributes:* younger physician age; female physician	**Intervention characteristics** *Cost:* lack of time **Outer setting** *Patient needs* *and* *resources:* patients' refusal to be diagnosed *Cosmopolitanism:* long wait for treatment referral **Inner setting** *Available resources:* lack of training
Geirsson (2005) [24] Sweden	Survey, 68 physicians, 193 nurses	**Outer setting** *Cosmopolitanism:* support services were readily available to refer patients to or better co-operation with the local community alcohol service **Inner setting** *Available resources:* quick and easy screening questionnaires and counselling materials were available; training programs for early intervention **Characteristics of individuals** *Knowledge and beliefs:* better practical skills in suitable interview technique	**Intervention characteristics** *Cost:* lack of time **Inner setting** *Available resources:* lack of training in counselling for reducing alcohol consumption; lack of counselling materials; lack of screening tools **Characteristics of individuals** *Knowledge and beliefs:* did not know how to identify problem drinkers
Gordon (2011) [56] USA	Survey, focus groups, 47 different health professionals (e.g. physicians and other staff)	**Intervention characteristics** *Adaptability:* computer-based methods for screening **Characteristics of individuals** *Knowledge and beliefs:* education	**Intervention characteristics** *Evidence strength and quality:* treatment did not work *Cost:* logistic problem **Outer setting** *Cosmopolitanism:* difficulty in patients’ referral **Inner setting** *Available resources:* lack of training; lack of alcohol screening tools
Gurugama (2003) [57] Sri Lanka	Survey, 105 physicians	**Outer setting** *Cosmopolitanism:* support from local services **Inner setting** *Available resources:* training **Characteristics of individuals** *Knowledge and beliefs:* rewarding to treat persons who misused alcohol *Self-efficacy:* confident in the ability to treat persons who misused alcohol	**Intervention characteristics** *Cost:* persons who misused alcohol presented major management problems; persons who misused alcohol were time-consuming to deal with **Inner setting** *Available resources:* lack of training to alcohol misuse **Characteristics of individuals** *Knowledge and beliefs:* lack of knowledge; negative attitudes towards persons misusing alcohol
Hanschmidt (2017) [58] France, Germany, Italy, Spain, and the UK	Survey, 2468 different health professionals (e.g. physicians, other staff)	N.A.	**Intervention characteristics** *Cost:* lack of time; too much effort needed **Inner setting** *Relative priority:* the low rating of importance of alcohol screening **Characteristics of individuals** *Knowledge and beliefs:* insufficient knowledge of screening tools; belief that regular screening was unnecessary
Holmqvist (2008) [59] Sweden	Survey, 1790 physicians, 2549 nurses	**Outer setting** *Patient needs and resources:* the patient had alcohol-related symptoms *Cosmopolitanism:* improved opportunities for referral **Inner setting** *Structural characteristics:* professional teamwork on problem drinkers *Access to knowledge and information:* improved greater supply of information materials **Characteristics of individuals** *Knowledge and beliefs:* improved knowledge about screening instruments; more knowledge about counselling techniques; more knowledge about how alcohol influences health**;** belief that asking patient’s alcohol consumption was their routine; the clear role of dealing with problem drinkers	**Intervention characteristics** *Cost:* lack of time **Characteristics of individuals** *Knowledge and beliefs:* uncertain how to ask; uncertain how to give advice; uncertain where to refer the patients
Hutchings (2006) [26] UK	Focus groups, 43 different health professionals (e.g. practice managers, receptionists, physicians)	**Intervention characteristics** *Adaptability:* targeted rather than universal screening, such as new patient registrations, general health checks, and particular types of consultations	**Intervention characteristics** *Evidence strength and quality:* uncertainty about the effectiveness of brief alcohol interventions *Cost:* workload or lack of time **Inner setting** *Structural characteristics:* lack of specialty staff *Organizational incentives and rewards:* lack of incentives *Available resources:* lack of space **Characteristics of individuals** *Knowledge and beliefs:* fear of harming the patient-physician relationship
Jakubczyk (2015) [60] Poland	Survey, 276 physicians	**Intervention characteristics** *Evidence strength and quality:* proven efficacy on early alcohol intervention **Outer setting** *Patient needs and resources:* patients’ requests for advice on alcohol consumption; patient’s willingness to pay for alcohol counselling *Cosmopolitanism:* provision of referral services *External policy and incentives:* public health education campaigns **Inner setting** *Organizational incentives and rewards:* quality assurance credits for providing early intervention; improving salary and working conditions *Available resources:* training was available; quick and easy screening questionnaire and counselling materials **Characteristics of individuals** *Knowledge and beliefs:* having a disease prevention model *Other personal attributes:* smaller number of patients seen by GP in an average week	**Intervention characteristics** *Cost:* lack of time **Outer setting** *Patient needs and resources:* patients’ refusal to change; no private insurance coverage for patients receiving alcohol counselling *External policy and incentives:* lack of government policy to support preventive medicine **Inner setting** *Organizational incentives and rewards:* lack of financial incentive *Available resources:* lack of screening tools; lack of counselling materials; lack of training **Characteristics of individuals** *Knowledge and beliefs:* having their own disease model rather than prevention model of alcohol problems; difficulty in identifying problem drinkers; belief that alcohol issue was not an important issue in general practice; belief that preventive health should be the patient’s responsibility but not theirs; having a liberal attitude to alcohol *Self-efficacy:* discomfort in dealing with alcohol issues *Other personal attributes:* physicians having alcohol problems
Johansson (2002) [61] Sweden	Survey, 65 physicians, 141 nurses	**Characteristics of individuals** *Knowledge and beliefs:* belief that health status was influenced by alcohol; perceived knowledge; perceived skills; belief that having the responsibility to help problem drinkers with early detection and brief intervention; belief that early detection was important; belief that working with alcohol-related problems worth the cost and effort; belief that anyone could develop alcohol problems	**Intervention characteristics** *Cost:* lack of time **Outer setting** *Patient needs and resources:* patient’s refusal for help **Characteristics of individuals** *Knowledge and beliefs:* belief that more tiring to take care of patients with drinking problems than other patients *Self-efficacy:* low self-efficacy in reducing patient’s alcohol consumption *Other personal attributes:* lower self-efficacy in nurses than physicians; nurses worried more about patients might react negatively to questions about alcohol
Johansson (2005) [62] Sweden	Focus groups, 13 physicians	**Outer setting** *Patient needs and resources:* patients had alcohol-related symptoms	**Outer setting** *Cost:* lack of time **Inner setting** *Compatibility:* doubt about the appropriateness of screening all patients **Characteristics of individuals** *Knowledge and beliefs:* fear of harming their relationship with the patient *Self-efficacy:* lack of self-efficacy
Johansson (2005) a[63] Sweden	Focus groups, 26 nurses	N.A.	**Intervention characteristics** *Cost:* lack of time **Characteristics of individuals** *Knowledge and beliefs:* fear of harming their relationship with the patient *Self-efficacy:* lack of self-efficacy
Kaariainen (2001) [64] Finland	Survey, 37 physicians, 32 nurses	**Characteristics of individuals** *Knowledge and beliefs:* belief that it was appropriate setting for early recognition and treatment	**Characteristics of individuals** *Knowledge and beliefs:* poor motivational skills; belief that discussing alcohol consumption was unacceptable *Self-efficacy:* low self-efficacy
Kaner (1999) [15] UK	Survey, 279 physicians	**Intervention characteristics** *Evidence strength and quality:* proven efficacy of SBI **Outer setting** *Patient needs and resources:* patients’ requests for health advice about alcohol consumption *Cosmopolitanism:* availability of appropriate support services to refer patients to *External policy and incentives:* public health campaigns make society more concerned about alcohol **Inner setting** *Organizational incentives and rewards:* salary conditions improved; training for early alcohol intervention recognized for CME; providing early alcohol intervention recognized for quality assurance credits *Available resources:* quick and easy counselling materials were available; training	**Intervention characteristics** *Cost:* lack of time **Outer setting** *Patient needs and resources:* patients’ unwillingness to receive help; no private insurance coverage for patients receiving alcohol counselling *External policy and incentives:* lack of government policy to support preventive medicine **Inner setting** *Organizational incentives and rewards:* lack of financial incentives *Available resources:* lack of training; lack of counselling materials **Characteristics of individuals** *Knowledge and beliefs:* having their own disease model rather than prevention model of alcohol problems; not sure how to identify problem drinkers; belief that preventive health should be the patient’s responsibility but not theirs; belief that general practice was not organized to do preventive counselling; belief that alcohol issue was not an important issue in general practice *Self-efficacy:* low self-efficacy
Kaner (2001) [65] Australia, Belgium, Bulgaria, Canada, France, Hungary, Italy, New Zealand, Norway, Poland, Portugal, Thailand, the UK	Survey, 2139 physicians	**Characteristics of individuals** *Knowledge and beliefs:* higher levels of alcohol-related education	N.A.
Kaner (2001) a[66] UK	Survey, 84 physicians, 12814 completed AUDIT screening questionnaires	**Outer setting** *Patient needs and resources:* more likely to give brief intervention when patients’ risk drinking status measured by total AUDIT score **Inner setting** *Available resources:* provision of training on brief intervention **Characteristics of individuals** *Other personal attributes:* patients were males, unemployed, and technically trained; GPs had longer average practice consultations; GPs in solo practice	**Characteristics of individuals** *Other personal attributes:* old patients (60-69 years old); students; university educated; unskilled workers
Kaner (2003) [67] UK	RCT, nurses in 156 general practices	**Inner setting** *Available resources:* provision of training	N.A.
Kersnik (2009) [19] Slovenia	Focus groups, 32 physicians	**Outer setting** *Cosmopolitanism:* professional institution, e.g., providing treatment suggestions *External policy and incentives:* SBI should be part of a national strategy or plan **Inner setting** *Organizational incentives and rewards:* financial support *Access to knowledge and information:* telephone support for questions regarding SBI implementation **Characteristics of individuals** *Knowledge and beliefs:* adequate knowledge and skills *Other personal attributes:* physician’s motivation	**Intervention characteristics** *Cost:* workload
Koopman (2008) [22] South Africa	Survey, 50 physicians	**Inner setting** *Available resources:* adequate training	**Intervention characteristics** *Cost:* lack of time **Outer setting** *Patient needs and resources:* patients’ refusal to take advice *External policy and incentives:* lack of support of government policy **Inner setting** *Organizational incentives and rewards:* difficulties in getting reimbursed for treating patients with alcohol problems *Available resources:* lack of training; lack of screening and counselling tools **Characteristics of individuals** *Knowledge and beliefs:* having their own disease model rather than prevention model of alcohol problems; didn’t know to identify problem drinkers; belief that general practice was not organized for preventive medicine *Self-efficacy:* low self-efficacy in helping patients reduce alcohol consumption; discomfort in asking patients’ alcohol drinking; belief that preventive health was patient’s responsibility
Kraus (2017) [68] Germany	Survey, 211 physicians	N.A.	**Intervention characteristics** *Cost:* lack of time **Outer setting** *Patient needs and resources:* patients’ denial **Characteristics of individuals** *Knowledge and beliefs:* lack of knowledge of appropriate alcohol screening instruments; alcohol was not an important risk factor
Le (2015) [69] USA	Survey, 210 physicians	N.A.	**Intervention characteristics** *Cost:* lack of time **Inner setting** *Organizational incentives and rewards:* lack of reimbursement *Available resources:* lack of adequate training **Characteristics of individuals** *Self-efficacy:* did not feel confident in helping at-risk drinkers
Lock (2002) [70] UK	Semi-structured interviews, 24 nurses	**Characteristics of individuals** *Knowledge and beliefs:* role legitimacy (belief that having the right to ask about patients’ drinking	**Characteristics of individuals** *Knowledge and beliefs:* confusion about the recommended sensible drinking limits; belief that drinking had social and coping functions
Lock (2004) [71] UK	128 nurses’ and patients’ data were collected and analysed	**Outer setting** *Patient needs and resources:* patients’ risk status as measured by AUDIT score was the most influential predictor for brief intervention *Other personal attributes:* male patients	N.A.
Marcell (2002) [72] USA	Survey, 1842 physicians	**Outer setting** *Cosmopolitanism:* physicians had places to refer patients **Characteristics of individuals** *Knowledge and beliefs:* positive beliefs about the importance of prevention; approved of early alcohol screening *Self-efficacy:* comfortable to manage alcohol patients *Other personal attributes:* female physician	N.A.
May (2006) [73] UK	Semi-structured interviews, 43 physicians	**Characteristics of individuals** *Knowledge and beliefs:* physicians already had their own strategies to ask about alcohol use using approaches incorporated over long-standing practice	N.A.
McAvoy (2001) [16] Australia, Canada, Denmark, France, Hungary, Italy, New Zealand, Norway, Poland, Russia UK	Semi-structured interview, 126 physicians	**Intervention characteristics** *Evidence strength and quality:* proven efficacy of early intervention **Outer setting** *Patient needs and resources:* patients’ requests for advice about alcohol consumption *External policy and incentives:* more societal concern about alcohol; government policy favoured preventive medicine; professional recognition of early intervention by medical bodies; policy making preventive medicine a higher status in the medical profession **Inner setting** *Organizational incentives and rewards:* financial reimbursement for training in early intervention; health scheme reimbursements; training in early intervention for hazardous alcohol consumption was recognized for continuing medical education credits; providing early intervention for hazardous alcohol consumption was recognized for quality assurance credits *Available resources:* quick and easy counselling techniques were available; quick and easy diagnostic questionnaires were available **Characteristics of individuals** *Knowledge and beliefs:* gained knowledge on alcohol in medical school	**Intervention characteristics** *Cost:* lack of time **Outer setting** *Patient needs and resources:* private health insurance did not reimburse patients for alcohol counselling; patient’s unwillingness to be asked; patient’s unwillingness for alcohol counselling *Cosmopolitanism:* lack of referral services *External policy and incentives:* lack of government policy support **Inner setting** *Organizational incentives and rewards:* lack of financial reimbursement or incentives in the contract *Available resources:* lack of training and education for early intervention in medical schools **Characteristics of individuals** *Knowledge and beliefs:* lack of counselling skills for reducing alcohol consumption
Miller (2006) [74] USA	Focus group, 18 different health professionals (e.g. medical assistants, nurses)	**Intervention characteristics** *Adaptability:* computer-based method for screening **Inner setting** *Structural characteristics:* teamwork *Relative priority:* prioritization	**Intervention characteristics** *Cost:* lack of time **Characteristics of individuals** *Knowledge and beliefs:* lack of knowledge of screening tools; fear of harming patient-physician relationship
Miquel (2018) [75] Spain	Survey, 867 physicians	**Characteristics of individuals** *Knowledge and beliefs:* higher levels of graduate education and postgraduate education in alcohol were more likely to provide screening	**Intervention characteristics** *Cost:* lack of time
Mules (2012) [76] New Zealand	Semi-structured interviews, 19 physicians	N.A.	**Intervention characteristics** *Cost:* lack of time **Outer setting** *Patient needs and resources:* patient dishonesty
Nevin (2002) [77] Canada	Survey, 75 physicians	N.A.	**Characteristics of individuals** *Self-efficacy:* lack of self-efficacy in counselling
Nygaard (2010) [18] Norway	Survey, 901 physicians	**Outer setting** *Cosmopolitanism:* access to specialized treatment for alcohol problems **Inner setting** *Organizational incentives and rewards:* reimbursement **Characteristics of individuals** *Knowledge and beliefs:* knowledge; better counselling skills *Other personal attributes:* male patients; young physicians	**Characteristics of individuals** *Knowledge and beliefs:* fear of harming their relationship with the patient *Self-efficacy:* low self-efficacy
Nygaard (2011) [78] Norway	Focus groups, 40 physicians	**Outer setting** *Cosmopolitanism:* referral services **Characteristics of individuals** *Knowledge and beliefs:* clear role of GPs in detecting alcohol problems	**Inner setting** *Compatibility:* doubt about the appropriateness to screen all patients; interruptions of the natural course of consultations **Characteristics of individuals** *Knowledge and beliefs:* difficulty in defining what is healthy drinking; fear of harming the physician-patient relationship
Owens (2000) [79] UK	Survey 101 nurses	N.A.	**Inner setting** *Available resources:* lack of training **Characteristics of individuals** *Knowledge and beliefs:* lack of knowledge and skills
Payne (2005) [80] Australia	Survey, 170 physicians	**Outer setting** *Patient needs and resources:* materials for patients *Cosmopolitanism:* referral services **Inner setting** *Available resources:* diagnosis materials for health professionals; availability of screening tools **Characteristics of individuals** *Knowledge and beliefs:* belief in preventive function of screening	N.A.
Peltzer (2008) [81] South Africa	Survey, semi-structured interviews, 214 different health professionals (i.e. clinic managers, nurses)	**Inner setting** *Structural characteristics:* teamwork *Relative priority:* less prioritized other health goals *Learning climate:* more chances to try and observe how to perform SBI *Available resources:* training **Characteristics of individuals** *Knowledge and beliefs:* belief that health status was influenced by alcohol **Process** *Reflecting and evaluating:* the feedback provided by the SBI trainers during their visits at the clinics	**Intervention characteristics** *Complexity:* perceived complexity *Cost:* workload **Characteristics of individuals** *Knowledge and beliefs:* belief that some people used alcohol for traditional purpose; belief that asking elderly about their drinking was a sign of disrepect
Poplas Susic (2010) [82] Slovenia	Focus groups, 32 physicians	N.A.	**Intervention characteristics** *Cost:* lack of time **Outer setting** *Patient needs and resources:* patients’ unwillingness to participate in SBI *External policy and incentives:* lack of government policy **Inner setting** *Compatibility:* interruptions of the natural course of consultations *Organizational incentives and rewards:* lack of funding; *Access to knowledge and information:* lack of guidelines **Characteristics of individuals** *Knowledge and beliefs:* lack of knowledge; inadequate counselling skills; disagreement over the recommended limits to the number of alcohol units per day/week; different interpretations regarding definitions of what constitutes an alcoholic beverage; fear of harming their relationship with the patient *Other personal attributes:* GPs’ alcohol drinking habits
Rapley (2006) [83] UK	Semi-structured interview, 43 physicians	**Outer setting** *Patient needs and resources:* patients actively seeking help **Characteristics of individuals** *Self-efficacy:* confidence in asking patients about their drinking	**Intervention characteristics** *Cost:* lack of time **Outer setting** *Cosmopolitanism:* lack of referral services **Inner setting** *Relative priority:* multiple problems of patients
Romero-Rodriguez (2019) [84] Spain	Survey, 1532 physicians and 220 nurses	**Characteristics of individuals** *Other personal attributes:* providers more likely to give advice: a nurse, female healthcare providers, providers aged 46–55 years	N.A.
Rush (1994) [85] Canada	Survey, 1235 physicians	**Characteristics of individuals** *Knowledge and beliefs:* belief that having the right to ask patients about their drinking; knowledge about drinking problems *Self-efficacy:* self-efficacy in giving advice	**Characteristics of individuals** *Knowledge and beliefs:* pessimistic attitudes towards problem drinkers; unwilling to work with problem drinkers; not feeling proud to work with problem drinkers; belief that it was unrewarding to work with problem drinkers
Rush (1995) [86] Canada	Focus groups, semi-structured interviews, 24 physicians	**Outer setting** *Cosmopolitanism:* available referral service **Characteristics of individuals** *Knowledge and beliefs:* belief that health status was influenced by health; belief that having the right to ask their patients about alcohol drinking; belief that having the responsibility to ask about patients’ alcohol consumption; belief that alcohol issue was an important issue in general practice	**Intervention characteristics** *Cost:* lack of time **Inner setting** *Compatibility:* doubt about the appropriateness of asking all patients *Available resources:* lack of screening materials **Characteristics of individuals** *Self-efficacy:* lack of confidence in helping patients reduce alcohol consumption *Other personal attributes:* female physicians **Process** *Executing:* lack of a systematic strategy
Seppanen (2012) [87] Finland	Survey, 2001 physicians (2002), 1610 physicians (2007)	**Characteristics of individuals** *Other personal attributes:* having a specialist’s licence in general practice or occupational health care; long experience as a GP	N.A.
Sharp (2011) [88] USA	Survey, 101 physicians	**Characteristics of individuals** *Self-efficacy:* self-efficacy in alcohol management skills *Other personal attributes:* long years of practice	N.A.
Slaunwhite (2015) [89] Canada	Survey, 67 physicians	**Characteristics of individuals** *Knowledge and beliefs:* belief that health status was influenced by alcohol	**Outer setting** *Cosmopolitanism:* lack of referral services
Spandorfer (1999) [90] USA	Survey, 131 physicians	N.A.	**Intervention characteristics** *Evidence strength and quality:* doubt about the effectiveness of treatment **Outer setting** *Cosmopolitanism:* lack of treatment resources
Tam (2013) [91] Australia	Focus group, 19 physicians	N.A.	**Outer setting** *Patient needs and resources:* unreliable patient alcohol use histories **Characteristics of individuals** *Knowledge and beliefs:* fear of harming their relationship with the patient
Van (2013) [92] South Africa	Survey, 77 physicians	N.A.	**Outer setting** *Cosmopolitanism:* lack of referral services **Inner setting** *Structural characteristics:* lack of multidisciplinary teams *Organizational incentives and rewards*: lack of medical funding *Available resources:* lack of in-patient facilities
Williams (2016) [27] USA	Semi-structured interviews, 32 different health professionals (e.g. clinical staff, providers, administrative staff)	**Characteristics of individuals** *Knowledge and beliefs:* belief that health status was influenced by alcohol; belief that alcohol issue was an important issue in general practice	**Outer setting** *Patient needs and resources:* patients’ discomfort; low interest of patients in seeking help *Cosmopolitanism:* limited treatment referral resources **Inner setting** *Goals and feedback:* lack of understanding of the goals of SBI *Available resources:* lack of training
Wilson (2011) [93] The UK	Survey, 282 physicians	**Intervention characteristics** *Evidence strength and quality:* proven efficacy *Patient needs and resources:* patients’ requests for advice **Outer setting** *Cosmopolitanism:* referral services were available *External policy*: public health campaigns **Inner setting** *Organizational incentives and rewards:* improving salary and working conditions *Available resources:* availability of easy and quick screening questionnaires and counselling materials; training; providing early invention recognized in quality assessment	**Outer setting** *Patient needs and resources:* patients’ refusal to take advice *Cost:* workload **Inner setting** *Organizational incentives and rewards:* lack of contractual incentives *Available resources:* lack of training; lack of counselling materials **Characteristics of individuals** *Knowledge and beliefs:* lack of knowledge to identify problem drinkers

Note: *AUDIT* Alcohol Use Disorder Identification Test, *CME* continuing medical education, *GP* general practitioner

### Practice of SBI

Although the analysis of the practice of SBI was not the main aim of this review, we tried to extract related information from the included studies and give a preliminary analysis in this area. Among the included studies, participants were asked about their current practice of SBI in 15 studies, and these studies were conducted in nine countries, i.e. Finland, the UK, Germany, the USA, France and South Africa, Sweden, Sri Lanka, and Canada (Additional file 3). The participants in these studies were all physicians, except in one study in which nurses were also included. There were two ways of measuring their practice: 1) had ever performed SBI; 2) performed SBI on a regular basis. The percentage of participants who reported that they had ever performed screening or brief intervention ranged from 45.0% to 100%. However, the percentage was much lower when it was on a regular basis, which ranged from 9.4% to 40.0%, except for one study with 75.0%. Regarding whether they had performed SBI, the highest rate was found in South Africa (100%), the UK (98.0%), the USA (95.0% and 84.0% in two studies), France (94.1%), and Germany (84.2%), whereas the lowest rate was found in Finland (45.0%). For regular basis, the highest rate was found in Canada (75.0%) and the UK (40%) whilst the lowest rate was found in Finland (9.4%) and Sri Lanka (15.0%).

### Facilitators and barriers of SBI implementation based on the CFIR

The following results are presented according to Table 2.

#### Intervention characteristics

Evidence strength was considered by primary healthcare providers when implementing SBI. About 74–81% of physicians in the UK and Poland agreed that the proven efficacy of early alcohol intervention was a facilitator of implementing SBI [15, 60, 93], whilst doubt about the effectiveness of brief interventions was cited as a barrier to implementing SBI by physicians or nurses in the USA, the UK, and Finland [21, 23, 26, 46, 56, 90]. For adaptability, physicians, nurses, and other health professionals (e.g. social workers, psychologists) in the USA, Catalonia, the Netherlands, Poland, Sweden, and the UK suggested that SBI could be adapted or refined to meet special needs, such as using computer-based methods for screening, targeted rather than universal screening (e.g. new patient registrations, general health checks, and particular types of consultations) [26, 56, 74]. Perceiving SBI to be more complex or difficult to implement was associated with poorer SBI implementation among nurses and clinic managers in clinics in South Africa [81]. For design quality, in one qualitative study, different health professionals (e.g. social workers, psychologists, nurses) in Brazil agreed that simple SBI techniques could facilitate SBI implementation [38]. Numerous studies reported some barriers related to the cost associated with implementing SBI. For example, the workload increased by implementing SBI or lack of time were frequently reported among GPs, nurses, and other health professionals [15, 16, 19–22, 24–26, 35, 37, 38, 41, 48, 50–55, 57–63, 68, 69, 74–76, 81–83, 86, 93] and about 36–76% of physicians or nurses thought that it would cause management or logistic problems [50–52, 56, 57].

Three constructs in this domain, intervention source, relative advantage, and trialability, were not studied.

#### Outer setting

For patient needs and resources, patients’ active role as a facilitator was revealed in numerous studies [13, 15, 16, 21, 47, 51, 59, 60, 62, 66, 71, 80, 83, 93]. For instance, about 52% of physicians, 50% of nurses, and 75% of health workers in Australia or Finland reported that patients’ willingness to be asked about their drinking consumption or receive advice was a facilitator [13, 47]. About 76–80% of physicians in Poland and the UK suggested that patients’ requests for health advice on alcohol consumption or self-motivation for seeking help were incentives for them to implement SBI [15, 16, 51, 60, 83, 93]. In addition, for two studies conducted in the UK, patients’ risk status as measured by Alcohol Use Disorder Identification Test (AUDIT) by physicians was the most influential predictor for brief intervention [66, 71]. On the other hand, patients’ negative reactions were cited as barriers to implementing SBI [15, 16, 22–24, 37, 41, 47–49, 54, 55, 60, 61, 68, 70, 76, 82, 91, 27, 93]. For example, about 39–96% of physicians in Sweden, the UK, Poland, and South Africa reported that patients’ refusal, unwillingness, low interests to take advice or receive help were barriers to implementing SBI [15, 16, 22, 47, 48, 54, 55, 60, 61, 82, 27, 93]. Several studies revealed other barriers to implementing SBI in the USA, Finland, France, Germany, including patients’ denial of alcohol misuse, dishonesty of alcohol consumption, and neglect of negative consequences caused by excessive alcohol consumption [23, 41, 48, 49, 54, 68, 76, 91].

For cosmopolitanism, available referral services were reported by physicians, nurses, and other health professionals as facilitators of implement SBI, such as the provision of addiction care and specialized treatment for alcohol problems [15, 18, 19, 24, 46, 50, 52, 57, 59, 60, 72, 78, 80, 86, 93]. For instance, about 59–94% of physicians and 57–83%% of nurses in Poland, Sweden, the UK, Sri Lanka, and the USA reported that access to local community alcohol teams, general support services (e.g. self-help or counselling), were facilitators of implementing SBI [15, 24, 46, 50, 52, 57, 59, 60, 72, 80, 93]. Regression analyses in one study conducted in Norway also showed that having places to refer patients to was significantly associated with physicians’ screening or brief intervention activity [18]. On the contrary, the lack of referral services was also cited as a barrier to implementing SBI [16, 38, 54–56, 83, 89, 90, 92, 27]. For instance, about 52–76%% of physicians in Canada and the USA mentioned that wait-lists were long and treatment services were limited [89, 90]. Moreover, univariate analyses showed that physicians in Sweden who infrequently addressed alcohol issues were more likely to be uncertain where to refer the patients [59].

Support from external policy was cited as an incentive to implement SBI [15, 16, 19, 60, 93]. About 65–82% of physicians in the UK and Poland suggested that implementation of SBI as part of a national strategy and more public health education campaigns make society more concerned about alcohol were enablers of SBI implementation [15, 60, 93]. However, lack of support from government policy was usually mentioned [15, 16, 22, 60, 82]. For instance, government policy that did not support preventive medicine was pointed out by 56-98% of physicians in South Africa, the UK, and Poland [15, 22, 60].

One construct, peer pressure, was not covered by previous studies.

#### Inner setting

For structural characteristics, teamwork or interprofessional cooperation in the delivery of SBI was suggested as a facilitator [38, 59, 74, 81]. In contrast, lack of staff, specialist support, or multidisciplinary team in primary care settings were cited as barriers [26, 41, 51, 92].

Concerning compatibility, doubt about the appropriateness of screening all patients and such activity causing interruptions of the natural course of consultations were reported by physicians in South Africa, Canada, and some European countries such as Norway, Slovenia, and the UK [42, 62, 78, 82, 86].

High prioritization of alcohol issue was cited as an enabler by physicians, nurses, and clinic managers in South Africa, and the USA [74, 81] whereas a low rating of the importance of alcohol matter, competing priorities, or patients with multiple problems were reported as barriers by physicians, nurses, social workers, psychologists and other health professionals in Brazil, France, Germany, Italy, Spain, and the UK [38, 58, 83].

Two types of organizational rewards and incentives reported as facilitators, including provision of financial reimbursements/ salary improvements, and training for early alcohol intervention recognized for quality assurance credits [15–19, 60, 93]. About 35–84% of physicians in Australia and some European countries considered that provision of financial reimbursements/salary improvements for carrying out early alcohol intervention, recognizing SBI training for continuing medical education (CME), or recognizing SBI for quality assurance credits were facilitators [15, 60, 93]. On the contrary, a lack of organizational incentives for SBI implementation was reported across countries [15, 16, 22, 26, 60, 69, 82, 92, 93]. For instance, about 31–90% of physicians in Poland, South Africa, the UK, and the USA reported that there was a lack of contractual incentives or the government health scheme did not reimburse their time spent on preventive medicine [15, 22, 60, 69, 93].

Regarding availability of resources, provision of SBI training was commonly discussed as a facilitator of implementation [13–15, 17, 22, 24, 45, 47, 50–53, 57, 60, 65–67, 80, 81, 93]. For example, about 42–90% of physicians, 90% of nurses in South Africa, Finland, the UK, Sweden reported provision of training in early alcohol intervention would encourage them to carry out screening or brief intervention [13, 15, 22, 24, 50–52, 57], and five studies conducted in 13 European, Asian, and American countries showed that receiving training in alcohol predicted more screening or intervention activities [14, 60, 65–67]. Nonetheless, lack of training was also frequently reported as a barrier in different studies [13, 15, 16, 22, 24, 39, 48, 50–52, 54–57, 60, 65, 69, 79, 27, 93]. For instance, about 32-98% of physicians and 75-90% of nurses in 19 Asian, American, African and European countries conducted by 12 studies reported that lack of training in detection in alcohol misuse, counselling in reducing alcohol consumption were the main barriers [13, 15, 22, 24, 39, 50–52, 57, 60, 65, 69]. Apart from training, provision of screening questionnaires or counselling materials was also frequently cited as a facilitator [15, 17, 24, 25, 60, 80, 93]. For instance, about 51-86% of physicians and 74% of nurses in Australia, Sweden, the UK, and Poland reported that provision of screening devices or counselling materials encouraged them to do early alcohol intervention [15, 16, 24, 35, 60, 80, 93]. On the contrary, lack of materials was reported as a barrier in different studies [15, 21–24, 56, 60, 86, 93]. For example, about 41–98% of physicians and 56–63% of nurses in the UK, Sweden, Poland, and South Africa reported that lack of screening devices or counselling materials discouraged them to do early alcohol intervention [15, 22, 24, 60, 93]. Furthermore, lack of other resources, such as space and in-patient facilities, were also reported as barriers by physicians, nurses, administrative staff, and practice managers in South Africa, the UK, and the USA [26, 92].

Regarding access to knowledge and information, easy access to clear guidelines related to implementing SBI was suggested [13, 47, 59]. Support calls responding to questions or problems that arose during SBI implementation were demonstrated to be effective in two multi-country studies [14, 40]. However, a lack of simple guidelines was reported by physicians and nurses in Finland and Slovenia [20, 82].

Four constructs in this domain were briefly discussed, i.e. culture, learning climate, goals and feedback, and leadership engagement. Regarding culture, one study conducted in Brazil reported that the organizational culture of alcohol use (e.g. often using alcohol during work-related celebrations) in the primary care service of the Military Police would hamper treating problem drinkers [38]. For learning climate, more chances to try and observe SBI were reported as facilitators by nurses and clinic managers in South Africa [81]. A lack of understanding of the goals of SBI was mentioned in one study in the USA [27]. A decrease in institutional support due to changes in leadership was also reported in one study conducted in Brazil [38].

In this domain, two constructs, networks and communications, and tension for change, were not studied.

#### Characteristics of individuals

Possession of knowledge and positive beliefs about the intervention were reported as facilitators by 35 studies [13, 14, 16–19, 24, 35, 39, 40, 45, 46, 49, 51–53, 55–57, 59–61, 64, 70, 72, 73, 75, 78, 80, 81, 83, 85, 86, 89, 27]. For example, familiarity with expert guidelines, perceived knowledge and skills of early alcohol intervention, and receiving higher levels of education training in alcohol were significantly associated with screening or intervention activity in one multi-country study and four others in Sweden, the USA, Spain, and Norway [18, 40, 55, 61, 75]. On the other hand, lack of knowledge, skills, or low awareness of alcohol problems were cited as barriers by 25 studies [13, 15, 16, 20–22, 24, 26, 35, 37, 41, 57–60, 64, 68, 70, 74, 78–80, 82, 91, 93]. For example, about 30–70% of physicians or 39–65% of nurses reported that they did not know how to identify problem drinkers or how to define heavy drinking [13, 15, 16, 22, 24, 57, 60, 93]. About 67–86% of physicians or 74–89% of nurses reported that they had insufficient knowledge about screening tools or counselling techniques [13, 16, 22, 41, 58]. For positive beliefs, screening or brief intervention activity was significantly associated with the belief of the importance of prevention or early alcohol intervention or having greater therapeutic commitment in working with alcohol problems in Sweden, the USA, France, and two multi-country studies [14, 40, 49, 61, 72]. However, many negative beliefs were also reported [15, 17, 18, 21–23, 25, 26, 39, 42, 46, 50–53, 57, 58, 60–64, 68, 70, 73, 74, 76, 78, 80–82, 85, 91]. The most common one was the belief that discussion about alcohol issues might harm the patient-physician relationship [18, 21, 25, 26, 37, 62, 63, 74, 78, 82, 91]. Other negative beliefs included treating problem drinkers was not rewarding, alcohol was not an important risk factor, preventive health should be the patients’ responsibility, and moderate use of alcohol was acceptable [15, 17, 22, 23, 39, 45, 49–52, 59, 71, 86].

Having high self-efficacy as an enabler was reported in different studies [13, 40, 43, 45, 50, 55, 57, 60, 72, 83, 85, 88]. For instance, studies conducted in the USA, Poland, and one multi-country study showed that having high self-efficacy in alcohol history taking or alcohol management skills in helping reduce patients’ alcohol consumption was associated with a higher number of interventions [40, 55, 60, 72, 88]. About 37–75% of physicians and 63% of nurses in the UK, Finland, or Sri Lanka reported that they were confident in asking, motivating, or influencing patients’ drinking [13, 50, 52]. However, low self-efficacy in inquiring about patients’ alcohol drinking, giving advice, or working in problem drinking in physicians or nurses were frequently reported [18, 22, 38, 42, 46, 51, 52, 60, 61, 69, 77, 86]. About 32–65% of physicians or 31–65% of nurses in the UK, South Africa, Sweden, Finland, or the USA reported that they did not feel confident in working with problem drinkers, counselling skills, and helping the patients to reduce drinking [15, 22, 46, 51, 52, 60, 61, 64, 69, 77].

For other personal attributes, many demographic characteristics were found to be positively associated with SBI implementation. These personal characteristics included male patients [18, 44, 66, 71], younger physician age [18, 55], smaller number of patients seen by GPs in an average week [60], GPs having longer average practice consultations [66], GPs in solo practice [66]. In contrast, other personal characteristics were found to be barriers, such as old patients aged 60–69 years [66], physicians having alcohol drinking habits or problems [60, 82], some nurses worrying more or having lower self-efficacy than physicians [61]. Nevertheless, there were mixed results that some studies showed that female healthcare provider was a facilitator [44, 55, 72, 84], whilst it was considered a barrier in one study [86].

In this domain, two constructs, i.e. individual stage of change, and individual identification with the organization were not covered by previous studies.

#### Process

There is a lack of studies in this domain. For executing, physicians reported that there was a lack of a systematic strategy in the clinic in one study conducted in Canada [86]. For reflecting and evaluating, the feedback provided by the SBI trainers during their visits at the clinics was found to be helpful in one study conducted in South Africa [81].

Two constructs, planning and engaging, had not been studied.

### Facilitators and barriers not covered by the CFIR

Facilitators that were not covered by the CFIR included medical documentation of patients’ alcohol drinking, reminders for providers to do SBI, and reminders for patients about their medical condition. Barriers that were not covered by the CFIR included stigma-related issues, conflicting messages in the society that drinking alcohol was acceptable and even beneficial to health, alcohol counselling involving family and wider social effects, and providers’ struggles in prevention versus treatment.

**Table 2:** Overall results of the findings using Consolidated Framework for Implementation Research

Domain/constructs	Facilitators of implementation	Population and range of sample size among different studies (*see the note below this table*)	Country (*see the note below this table*)	Reference	Barriers of implementation	Population and range of sample size among different studies (*see the note below this table*)	Country (*see the note below this table*)	Reference
**Intervention characteristics**								
Intervention source	–	–	–	–	–	–	–	–
Evidence strength and quality	Proven efficacy of SBI	P(4): 126-282	AU(1) CA(1) DK(1) FR(1) GB(3) HU(1) IT(1) NO(1) NZ(1) PO(1) RU(1)	[15, 16, 60, 93]	Doubt about the effectiveness of SBI	P(4): 75–131 DHP(2): 43–47	FI(2) GB(2) US(2)	[21, 23, 26, 46, 56, 90]
Relative advantage	–	–	–	–	–	–	–	–
Adaptability	Targeted rather than universal screening, such as new patient registrations, general health checks, and particular types of consultations	DHP(1): 43	GB(1)	[26]	–	–	–	–
	Computer-based methods for screening	DHP(2): 18-47	US(2)	[56, 74]	–	–	–	–
Trialability	–	–	–	–	–	–	–	–
Complexity	–	–	–	–	Perceiving SBI as a complex intervention	DHP(1): 214	ZA(1)	[81]
Design quality and packaging	Simplicity of SBI techniques	DHP(1): 79	BR(1)	[38]	–	–	–	–
Cost	–	–	–	–	Workload or lack of time	P(32):13–2377 N(6):19–2549 DPH(4):18–2468	AU(1) BR(1) CA(2) DE(2) DK(1) ES(3) FI(2) FR(2) GB(9) HU(1) IT(2) LK(1) NO(1) NZ(2) PO(2) RU(1) SE(6) SI(2) TR(1) US(5) ZA(1)	[15, 16, 19–22, 24–26, 37, 38, 41, 48, 50–55, 57–63, 68, 69, 74–76, 82, 83, 86, 93]
	–	–	–	–	Causing management or logistic problems	P(4): 81–2377 N(1): 196DHP(1): 47	GB(3) LK(1) US(1)	[50–52, 56, 57]
	–	–	–	–	Considerable resources or too much effort needed	P(1): 24 DHP(1): 2468	DE(1) DK(1) ES(1) FR(1) GB(1) IT(1)	[42, 58]
**Outer setting**								
Patient needs and resources	Willingness to be asked about their drinking consumption, receive adv, or pay for alcohol counselling	P(2): 84–276 N(1): 167 DHP(1): 47	AU(1) FI(1) PO(1)	[13, 47, 60]	Refusal, unwilling, or low interest to take advice or receive help	P(11): 32–853 N(2): 47–141 DHP(2): 32–47	AU(2) CA(1) DK(1) ES(1) FR(1) GB(3) HU(1) IT(1) NZ(1) NO(1) PO(2) RU(1) SE(1) SI(1) TR(1) US(3) ZA(1)	[15, 16, 22, 37, 47, 48, 54, 55, 60, 61, 82, 27, 93]
	Request for health advice on alcohol consumption or self-motivation for seeking help	P(6): 43–2377	AU(1) CA(1) DK(1) FR(1) GB(4) HU(1) IT(1) NZ(1) NO(1) PO(2) RU(1)	[15, 16, 51, 60, 83, 93]	Denial of alcohol misuse	P(5): 35–211	DE(1) FI(1) FR(1) GB(1)US(2)	[23, 41, 49, 54, 68]
	Showing alcohol-related symptoms	P(3):13–1790 N(1): 2549	FI(1) SE(2)	[21, 59, 63]	Dishonesty of alcohol consumption or unreliable patient alcohol use histories	P(2): 19	AU(1) NZ(1)	[76, 91]
	Clear reason for consultation of patients	P(1): 35	FI(1)	[21]	Neglect of negative consequences caused by excessive alcohol consumption	P(1): 60 N(1): 47	ES(1)	[48]
	Risk status as measured by AUDIT score	P(1): 84 N(1): 128	GB(2)	[66, 71]	Private health insurance did not reimburse patients for alcohol counselling	P(3): 126–279	AU(1) CA(1) DK(1) FR(1) GB(2) HU(1) IT(1) NZ(1) NO(1) PO(2) RU(1)	[15, 16, 60]
	Materials for patients	P(1): 170	AU(1)	[80]	Discomfort when talking about alcohol issues	DHP(1): 32	US(1)	[27]
Cosmopolitanism	Referral services were available, such as provision of addiction care, specialized treatment for alcohol problems, access to local community alcohol teams, general support services (e.g. self-help or counselling)	P(15): 24–1842 N(2): 193–2549	AU(1) CA(1) GB(5) LK(1) NO(2) PO(1) SE(2) SI(1) US(1)	[15, 18, 19, 24, 46, 50, 52, 57, 59, 60, 72, 78, 80, 86, 93]	Lack of referral services or difficulty in patients’ referral	P(7): 42–853 DHP(4): 32–79	AU(1) BR(1) CA(2) DK(1) FR(1) GB(2) HU(1) IT(1) NL(1) NZ(1) O(1) PO(1) RU(1) US(5) ZA(1)	[16, 38, 55, 56, 83, 89, 90, 92, 27]
Peer pressure	–	–	–	–	–	–	–	–
External policy and incentives	Implementation of SBI using a national alcohol strategy;	P(1): 32	SI(1)	[19]	Lack of government policy to support preventive medicine	P(5): 32–279	AU(1) BR(1) CA(1) DK(1) FR(1) GB(2) HU(1) IT(1) NZ(1) NO(1) PO(2) RU(1) SI(1) ZA(1)	[15, 16, 22, 60, 82]
	Public health education campaigns make society more concerned about alcohol;	P(5\4): 126–282	AU(1) CA(1) DK(1) FR(1) GB(3) HU(1) IT(1) NZ(1) NO(1) PO(2) RU(1)	[15, 16, 60, 93]	–	–	–	–
	Policy making preventive medicine a higher status in the medical profession;	P(1): 126	AU(1) CA(1) DK(1) FR(1) GB(3) HU(1) IT(1) NL(1) NZ(1) NO(1) PO(1) RU(1)	[16]	–	–	–	–
	Professional recognition of early intervention by medical bodies	P(1): 126	AU(1), CA(1) DK(1) FR(1) GB(1) HU(1) IT(1) NL(1) NZ(1) NO(1) PO(1) RU(1)	[16]	–	–	–	–
**Inner setting**								
Structural characteristics	Teamwork or interprofessional cooperation in the delivery of SBI	P(1): 1790 N(1): 2549 DHP(3): 18–214	BR(1) SE(1) US(1) ZA(1)	[38, 59, 74, 81]	Lack of staff, specialist support or multidisciplinary team	P(3): 41–2377 DHP(1): 43	GB(2) US(1) ZA(1)	[26, 41, 51, 92]
Networks and communications	–	–	–	–	–	–	–	–
Culture	–	–	–	–	Organizational culture about alcohol use	DHP(1): 79	BR(1)	[38]
Tension for change	–	–	–	–	–	–	–	–
Compatibility	–	–	–	–	Interruptions of the natural course of consultations	P(3): 24–40	DK(1) NO(1) SI(1)	[42, 78, 82]
	–	–	–	–	Doubt about the appropriateness of screening all patients	P(3): 24–40	CA(1) NO(1) SE(1)	[62, 78, 86]
Relative priority	Prioritization of alcohol issues	DHP(2): 18–214	US(1) ZA(1)	[74, 81]	Low rating of importance of alcohol screening, patients with multiple problems or other competing priorities	P(1): 43 DHP(2): 79–2468	BR(1) DE(1) ES(1) GB(2) IT(1)	[38, 58, 83]
Organizational incentives and rewards	Financial support/ incentives/reimbursements, such as improving salary conditions, health scheme reimbursements	P(8): 32–2345	AU(1) CA(1) CZ(1) DK(1) ES(1) FR(1) GB(4) HU(1) IT(2) NL(1) NO(2) NZ(1) PO(3) PT(1) RU(1) SI(2)	[15–19, 60, 93]	Lack of financial support, incentives, reimbursement, funding, such as Contractual incentives, time spent on treating alcohol patients	P(8): 32–282 DHP(1): 43	AU(1) CA(1) K(1) FR(1) GB(4) HU(1) IT(1) NO(1) NZ(1) PO(2) RU(1) SI(1) US(1) ZA(2)	[15, 16, 22, 26, 60, 69, 82, 92, 93]
	Training in early alcohol intervention recognized for continuing medical education credits	P(2): 126–279	AU(1), CA(1) DK(1), FR(1) GB(2) HU(1) IT(1) NZ(1) NO(1) PO(1) RU(1)	[15, 16]	–	–	–	–
	Providing early alcohol intervention recognized for quality assurance credits	P(3): 276–282	GB(2), PO(1)	[15, 60, 93]	–	–	–	–
Goals and feedback	–	–	–	–	Lack of understanding of the goals of SBI	DHP(1): 32	US(1)	[27]
Learning climate	More chances to try and observe SBI	DHP(1): 214	ZA(1)	[81]	–	–	–	–
Leadership engagement	–	–	–	–	Variability of the institutional support due to changes in leadership	DHP(1): 79	BR(1)	[38]
Available resources	Training	P(15): 15–2377 N(3): 167–196 DHP(2): 47–214	AU(5) BE(2) BG(1) CA(2) CZ(1) ES(1) FI(1) FR(1) GB(11) U(1) IT(2) LK(1) NL(1) NO(1) NZ(1) PO(3) PT(2) SE(1) SI(1) TH(1) ZA(2)	[13–15, 17, 22, 24, 45, 47, 50–53, 57, 60, 65–67, 80, 81, 93]	Lack of training in detection in alcohol misuse, counselling in reducing alcohol consumption	P(17): 42–2377 N(4): 47–196 DHP(2): 32–47	AU(2) BE(1) BG(1) CA(2) DK(1) ES(1) FI(1) FR(2) GB(7) HU(2) IT(2) LK(1) NZ(2) NO(2) PO(3) PT(1) RU(1) SE(1) TH(1) US(6) ZA(1)	[13, 15, 16, 22, 24, 39,50–52, 54–57, 60, 65, 69, 79, 27, 93]
	Screening and counselling materials were available	P(9): 13–2345 N(1): 193	AU(3) CA(1) CZ(1) DK(1) ES(1) FI(1) FR(1) GB(4) HU(1) IT(2) NL(1) NO(1) NZ(1) PO(3) PT(1) RU(1) SE(2) SI(1)	[15–17, 24, 25, 35, 60, 80, 93]	Lack of screening devices or counselling materials	P(8): 24–282 N(1): 193 DHP(1): 47	CA(1) FI(2) GB(2) SE(1) PO(1) US(1) ZA(1)	[15, 21–24, 56, 60, 86, 93]
	–	–	–	–	Lack of space and in-patient facilities	P(1): 77 DHP(2): 32–47	GB(1) US(1) ZA(1)	[26, 92]
Access to knowledge and information	Easy access to clear guidelines or information related to implementing SBI	P(2): 84–1790 N(2): 167–2549 DHP(1): 47	AU(1) FI(1) SE(1)	[13, 47, 59]	Lack of guidelines	P(2): 18–32 N(1): 19	FI(1) SI(1)	[20, 82]
	Support calls responding to questions or problems that arose during SBI	P(2): 632–1300	AU(2) BE(2) CA(2) FR(1) GB(2) IT(1) NO(1) NZ(1) PT(1)	[14, 40]	–	–	–	–
**Characteristics of individuals**								
Knowledge and beliefs about the intervention	Knowledge: knowledge, qualification, or education level of alcohol medicine	P(8): 50–2345 DHP(1): 47	AU(2) BE(1) CA(3) CZ(1) DK(1) ES(2) FR(2) GB(5) HU(1) IT(3) NL(1) NZ(2) NO(2) PO(2) PT(2) RU(1) SE(1) SI(1) US(1)	[16, 17, 40, 46, 53, 56, 59, 75, 85]	Knowledge: confusion regarding the definition of early-phase heavy drinking and problem drinkers, the recommended sensible drinking limits, or what is health drinking	P(12): 18–282 N(5): 19–193	AU(1) CA(1) DK(1) FI(2) FR(1) GB(5) HU(1) IT(1) LK(1) NO(2) NZ(1) PO(2) RU(1) SE(1) SI(1) TR(1) ZA(1)	[13, 15, 16, 20, 22, 24, 37, 57, 60, 70, 78, 79, 82, 93]
	Familiarity with expert guidelines	P(1): 853	US(1)	[55]	Insufficient knowledge of screening tools, intervention techniques, counselling skills	P(10): 19–1790 N(3): 32–2549 DHP(2): 18–2468	AU(2) CA(1) DE(2) DK(1) FI(2) ES(1) FR(2) GB(2) HU(1) IT(2) NZ(1) NO(1) PO(1) RU(1) SE(1) SI(1) TR(1) US(2) ZA(1)	[13, 16, 22, 37, 41, 58, 59, 64, 68, 74, 82, 91]
	knowledge of alcohol screening or brief intervention;	P(6): 15–1790 N(2): 28–2549	AU(1) ES(1) NL(1) NO(1) PO(1) SE(3) SI(1)	[18, 19, 45, 59, 61]	Having their own disease model rather than prevention model of alcohol problems	P(4): 50–2345	ES(1) CZ(1) GB(2) IT(1) NL(1) PO(2) PT(1) SI(1) ZA(1)	[15, 17, 22, 60]
	already had their own strategies in asking patients about their alcohol drinking;	P(1): 43	GB(1)	[73]	Low awareness of alcohol problems	P(2): 35–170 DHP(1): 43	AU(1) FI(1) GB(1)	[21, 26, 80]
	practical skills in interviewing or counselling technique	P(2): 68–1790 N(2): 193–2549	NO(1) SE(2)	[18, 24, 59]				
	Beliefs: the belief that having the responsibility to ask about patient's alcohol consumption	P(5): 24–1790 N(2): 141–2549	CA(1) FI(1) NO(2) SE(2)	[35, 59, 61, 78, 86]	Beliefs: the belief that discussion about alcohol issues might harm the patient-physician relationship	P(8): 13–901 N(1): 26 DHP(2): 18–43	AU(1) FI(1) GB(1) NO(2) SE(3) SI(1) TR(1) US(1)	[18, 21, 25, 26, 37, 62, 63, 74, 78, 82, 91]
	The belief that having the right to ask patients about their drinking	P(4): 24–1235 N(1): 24	CA(2) GB(3) US(1)	[39, 46, 53, 70, 85, 86]	The belief that alcohol was not an important risk factor	P(2): 35–211	DE(1) FI(1)	[23, 68]
	Greater therapeutic commitment to working with alcohol problems	P(3): 101–1300	AU(2) BE(2) CA(2) FR(2) GB(2) IT(1) NZ(1) NO(1) PT(1)	[14, 40, 49]	The belief that drinking was a personal rather than a medical responsibility	P(5): 50–2345	CZ(1) ES(1) GB(3) IT(1) NL(1) PO(3) PT(1) SI(1) ZA(1)	[15, 17, 22, 53, 60]
	The belief that health status was influenced by alcohol	P(3): 24–67 N(1): 141 DHP(1): 214	CA(2) SE(1) US(1) ZA(1)	[61, 81, 86, 89, 27]	The belief that alcohol issue was not an important issue in general practice	P(3): 135–279	GB(1) PO(1) TR(1)	[15, 60, 37]
	The belief that it was rewarding to treat patients with alcohol use disorder	P(1): 105	LK(1)	[57]	The belief that general practice was not organized for preventive medicine	P(2): 50–279	GB(1) ZA(1)	[15, 22]
	The belief that it was rewarding to treat patients with alcohol use disorder	P(1): 105	LK(1)	[57]	The belief that it was not rewarding to work with drinkers	P(6): 71–2377	CA(1) GB(4) US(1)	[39, 46, 50–52, 85]
	The belief in preventive function of screening	P(4): 65–1842 N(1): 141	AU(1) PO(1) SE(1) US(1)	[60, 61, 72, 80]	The belief that discussing alcohol consumption was unacceptable	P(1): 37 N(1): 32	FI(1)	[64]
	The belief that anyone could develop alcohol problems	P(1): 65 N(1): 141	SE(1)	[61]	The belief that regular screening was unnecessary	DHP(1) 2468	DE(1) ES(1) FR(1) GB(1) IT(1)	[58]
	The belief that general practice was an appropriate setting or alcohol issue was an important issue in general practice	P(6): 24–2377 N(3): 32–196 DHP(1): 32	CA(1) FI(2) GB(3) US(1)	[13, 51–53, 64, 86, 27]	The belief that moderate use of alcohol was acceptable or it had social or coping function	P(2): 35–276 N(1): 14	FI(1) GB(1) PO(1)	[23, 60, 70]
					Doubt about the rationale in screening in young people	P(1): 24	DK(1)	[42]
					The belief that some people used for traditional purpose	DHP(1): 214	ZA(1)	[81]
					The belief that asking elderly about their drinking was a sign of disrespect	DHP(1): 214	ZA(1)	[81]
					Other negative/pessimistic attituded towards alcohol patients such as not feeling proud, unwilling to work with drinkers, more tiring to take care of Patients with alcohol problem than other patients	P(4): 50–1235 N(1): 141	CA(1) GB(1) LK(1) SE(1)	[53, 57, 61, 85]
Self-efficacy	Self-efficacy in alcohol history taking;	N(1): 196	US (1)	[55]	Low self-efficacy in inquiring about patients’ alcohol drinking, giving advice, counselling patients	P(4): 24–75	CA(1) DK(1) GB(1) ZA(1)	[22, 42, 46, 77]
	Confident in alcohol management skills or in asking, giving advice, motivating or influencing patients’ drinking	P(12): 15–2345 N(1): 167 DHP(1): 746	AU(2) BE(1) CA(2) ES(1) FI(1) FR(1) GB(4) IT(1) LK(1) NL(1) NO(1) NZ(1) PO(2) PT(1) SE(1) US(2)	[13, 40, 43, 45, 50, 57, 60, 72, 83, 85, 88]	Not confident or discomfort in working in alcohol issues (e.g. Establishing rapport with patients) or in helping patients reduce alcohol consumption	P(13): 15–2377 N(3): 19–141 DHP(1): 79	BR(1) CA(1) DK(1) FI(2) GB(3) NO(1) PO(1) SE(3) US(1) ZA(1)	[15, 18, 20, 22, 38, 42, 51, 52, 60–64, 69, 86]
Individual stage of change	–	–	–	–	–	–	–	–
Individual identification with the organization	–	–	–	–	–	–	–	–
Other personal attributes	Male patients	P(3): 58–901 N(1): 128	DE(1) GB(2) NO(1)	[18, 44, 66, 71]	University educated or old patients (60–69 years old)	P(1): 84	GB(1)	[66]
	Unemployed patients	P(1): 84	GB(1)	[66]	Physicians had alcohol drinking habits or problems	P(2): 32–276	PO(1) SI(1)	[60, 82]
	Younger physician age	P(2): 853–901	NO(1) US(1)	[18, 55]	Some nurses worried more or had lower self-efficacy than physicians	P(1): 65 N(1): 141	SI(1)	[61]
	Female healthcare providers	P(4): 58–1842 N(1): 228	DE(1) ES(1) US(2)	[44, 55, 72, 84]	Lack of motivation of physicians	P(1): 312	US(1)	[39]
	Longer years of practice	P(2): 101–3611	FI(1) US(1)	[87, 88]	Female physicians	P(1): 24	CA(1)	[86]
	Physicians (asking about alcohol use)	P(1): 65 N(1): 141	SE(1)	[61]	–	–	–	–
	Nurses (provided advice for reducing alcohol use)	P(1): 1543 N(1): 228	ES(1)	[84]	–	–	–	–
	Smaller number of patients seen by GP in an average week	P(1): 276	PO(1)	[60]	–	–	–	–
	Longer average practice consultations	P(1): 84	GB(1)	[66]	–	–	–	–
	Solo practice	P(1): 84	GB(1)	[66]	–	–	–	–
	Physician’s motivation or interest in alcohol issues	P(2): 32–71	GB(1) SI(1)	[19, 46]	–	–	–	–
	Having a specialist licence in general practice or occupational health care	P(2): 1909–3611	FI(2)	[36, 87]	–	–	–	–
**Process**								
Planning	–	–	–	–	–	–	–	–
Engaging	–	–	–	–	–	–	–	–
Executing	–	–	–	–	Lack of a systematic strategy	P(1): 24	CA(1)	[86]
Reflecting and evaluating	The feedback provided by the SBI trainers during their visits at the clinics	DHP(1): 214	ZA(1)	[81]	–	–	–	–

Note:

1. Abbreviations for populations: *P* physicians, *N* nurses, *DHP* different health professionals

2. The number in the bracket in Population and Country indicated the number of studies, and the number on the right-hand side in Population indicated the range of numbers of participants

3. Abbreviations for country names: *AU* Australia, *BE* Belgium, *BG* Bulgaria, *BR* Brazil, *CA* Canada, *CZ* Czech Republic, *DE* Germany, *DK* Denmark, *ES* Spain, *FI* Finland, *FR* France, *GB* United Kingdom of Great Britain and Northern Ireland, *HU* Hungary, *IT* Italy, *LK* Sri Lanka, *NL* Netherlands, *NZ* New Zealand, *NO* Norway, *PO* Poland, *PT* Portugal, *RU* Russia, *SE* Sweden, *SI* Slovenia, *TH* Thailand, *TR* Turkey, *US* the United States of America, *ZA* South Africa

## Discussion

In this systematic review, we sought to identify facilitators and barriers of SBI implemented by health professionals in primary care settings. We used the CFIR framework to analyse and summarize the facilitators and barriers.

We found that the practice rate of SBI was low in most countries on a regular basis. Among these countries, Finland, England, and France have participated in the WHO Phase IV implementation project (World Health Organization Collaborative Project on Identification and Management of Alcohol-Related Problems in Primary Health Care) [94]. Although the practice rate has increased since the commencement of this project, e.g. Finland [87], their practice rate is still below the satisfactory level. This may be due to social contextual factors. For instance, in Finland, alcohol problems used to be handled mostly by social welfare authorities and the police, and the disease model was adopted only later [21]. For future studies, researchers should investigate the specific contextual facilitators and barriers, and devise targeting strategies to help health professionals improve the practice of SBI in primary care settings. Nonetheless, the analysis of the practice of SBI was not the main aim of this review. Future work on the methodology of searching for articles specifically studying the current practice of SBI is recommended and a separate review and in-depth analysis will bring a more thorough discussion in this area.

The most common facilitators were knowledge and positive beliefs about SBI (characteristics of the individuals) and available resources (inner setting), whilst the most common barriers were cost related to implementing SBI (intervention characteristics), negative beliefs and the lack of knowledge (characteristics of the individuals), and the lack of self-efficacy (characteristics of the individuals). Notably, knowledge or lack of knowledge was cited as the most common facilitator or barrier. Knowledge as a facilitator contains several aspects, such as education on how alcohol influences health, familiarity with screening instruments, and counselling or intervention skills [24, 59, 85]. On the other hand, health professionals expressed difficulty in defining what is healthy drinking in different studies [13, 20, 78, 82]. It was argued that many primary healthcare professionals still seemed to have the outdated dichotomous model of alcohol drinkers, i.e. those with alcohol dependence and moderate drinkers [20]. They should be informed that a more complex spectrum dividing alcohol problems into more detailed subgroups has been suggested, such as hazardous and harmful drinking in addition to alcohol dependence [20]. Training was the most common facilitator in the construct—available resources. Training is important for health professionals to acquire knowledge and practice skills required to perform screening and brief intervention. Under the construct cost, time cost appeared to be a prominent barrier. In most primary care settings, most physicians and nurses have to carry out a predetermined number of consultations, tasks, and assessments per day. They have limited time for each patient. They need to set priorities for screening conditions that they have tools and knowledge, or based on patients’ requests [21, 59]. Lack of self-efficacy is another important barrier. One of the main reasons may be the lack of guidelines about what they should do to alcohol drinkers [20, 82]. Allowing the professionals to apply simple guidelines creatively and select what was the most appropriate for their patients was related to higher self-efficacy [20, 46].

Most of the factors of SBI implementation identified in this systematic review are modifiable. There are a host of implementation strategies developed by implementation researchers for modifying factors to implement an intervention. For instance, Expert Recommendations for Implementing Change (ERIC) comprises a list of 73 implementation strategies [95–97]. The CFIR-ERIC Matching tool was developed which provides a list of implementation strategies to consider based on the CFIR-based barriers/facilitators [98, 99]. For instance, if the barrier, lack of self-efficacy, is selected, strategies such as conducting ongoing training, making training dynamic, providing ongoing consultations will be provided. For another example, if adaptability is selected, strategies such as conducting local needs assessment, identifying early adopters, and making tailoring strategies will be provided.

Moreover, when looking at how factors were studied according to the constructs in the CFIR framework, we observed that factors related to the inner setting and characteristics of individuals were extensively studied whilst the process received the least attention. Constructs in the inner setting and characteristics of individuals mainly focus on the assets of the organization and individuals, respectively. Researchers have put lots of effort into studying how the assets, such as available resources, knowledge, and training, affected the implementation of alcohol screening and brief intervention. Nonetheless, the role of the dynamic process of implementing SBI is generally neglected in previous studies, such as the process of planning, how different types of leaders engage in the implementation, as well as how to execute and evaluate the implementation process. It is conceivable that the consistently low uptake rate of SBI might be due to the lack of understanding of factors related to the implementation process. Therefore, future studies should examine the execution and processes of the SBI implementation.

To our knowledge, the CFIR was used by one systematic review to synthesize factors of implementing enhanced recovery pathways [100]. Similar to our findings, that systematic review suggested that more effort should be put into the implementation process in future studies. This conveys a message that the neglect of the implementation process may be one of the main reasons for the low uptake rate of many evidence-based practices in real settings. Therefore, using the CFIR to do systematic reviews of facilitators and barriers can help us gain a thorough understanding of implementation research . 

There are three limitations in this systematic review. Firstly, we could not investigate whether there is a difference in facilitators or barriers between different types of health professionals, such as physicians and nurses, due to a relatively small number of studies involving nurses in this review. Secondly, different health systems or settings might induce different facilitators or barriers. Nevertheless, there is not enough information in the studies for stratification into different health systems or to the public or private sector. Since administration, culture, and management might be very different in different systems or sectors, it is possible that factors affecting the implementation of SBI might also be different. Thirdly, although the CFIR is comprehensive, it might not be able to cover some facilitators/barriers (e.g. stigma).

## Conclusions

Most literature focused on various kinds of available assets to implement SBI. To promote the spread of SBI implementation, more high-quality studies on the implementation process are needed. This systematic review could serve as a reference framework for health authorities to devise strategies for improving the implementation of SBI in primary care settings.

## Supplementary Information

**Additional file 1.**

**Additional file 2.**

**Additional file 3.**

## Data Availability

All data generated or analysed during this study are included in this article (and its supplementary information files).

## Abbreviations

AUDIT: Alcohol Use Disorder Identification Test
CFIR: Consolidated Framework for Implementation Research
CME: Continuing medical education
ERIC: Expert Recommendations for Implementing Change
GP: General practitioner
MMAT: Mixed-Method Appraisal Tool
RCT: Randomized controlled trial
SBI: Alcohol screening and brief intervention
WHO: World Health Organization
