# Supplementary material for: Using Consolidated Framework for Implementation Research to investigate facilitators and barriers of implementing alcohol screening and brief intervention among primary care health professionals: a systematic review
Source: Implement Sci. 2021 Nov 20;16:99. doi: 10.1186/s13012-021-01170-8 (PMC8605518; doi:10.1186/s13012-021-01170-8)
Supplement: Supplementary file 1 — Additional file 1. [file 13012_2021_1170_MOESM1_ESM.docx]

Additional file 1 Search strategy

| Steps | Search terms |
| --- | --- |
| 1 | alcohol* |
| 2 | drink* |
| 3 | interven* |
| 4  5 | screen*  manag* |
| 6 | 1 AND 3 |
| 7  8 | 1 AND 4  1 AND 5 |
| 9 | 2 AND 3 |
| 10  11 | 2 AND 4  2 AND 5 |
| 12 | 6 OR 7 OR 8 OR 9 OR 10 OR 11 |
| 13 | facilitat* OR barrier* OR evaluat* OR obstacle* OR predict* OR factor* OR view* OR perception* OR experience* OR attitude* OR opinion* OR strateg* OR enabl* OR difficult* |
| 14 | clinician* OR doctor* OR physician* OR practitioner* OR professional* OR worker* OR nurse* OR GP* |
| 15 | “primary care” OR “primary health care” OR “primary health*” OR “general practi*” |
| 16 | 12 AND 13 AND 14 AND 15 |
