# Supplementary material for: Using Consolidated Framework for Implementation Research to investigate facilitators and barriers of implementing alcohol screening and brief intervention among primary care health professionals: a systematic review
Source: Implement Sci. 2021 Nov 20;16:99. doi: 10.1186/s13012-021-01170-8 (PMC8605518; doi:10.1186/s13012-021-01170-8)
Supplement: Supplementary file 2 — Additional file 2. [file 13012_2021_1170_MOESM2_ESM.docx]

Additional file 2 Quality assessment of included studies by Mixed Methods Appraisal Tool (MMAT) – Version 2011

| Criteria |  | QND  n=47  (%) | QNR  n=2  (%) | QL  n=22  (%) | MM  n=3  (%) |
| --- | --- | --- | --- | --- | --- |
| QND1. Is the sampling strategy relevant to address the quantitative research question (quantitative aspect of the mixed methods question)? | Yes | 98 |  |  | 100 |
|  | No | 2 |  |  | 0 |
|  | Can’t tell | 0 |  |  | 0 |
| QND2. Is the sample representative of the population under study? | Yes | 74 |  |  | 33 |
|  | No | 26 |  |  | 67 |
|  | Can’t tell | 0 |  |  | 0 |
| QND3. Are measurements appropriate (clear origin, or validity known, or standard instrument)? | Yes | 85 |  |  | 100 |
|  | No | 0 |  |  | 0 |
|  | Can’t tell | 15 |  |  | 0 |
| QND4. Is there an acceptable response rate (60% or above)? | Yes | 49 |  |  | 67 |
|  | No | 40 |  |  | 33 |
|  | Can’t tell | 11 |  |  | 0 |
| QNR1. Is there a clear description of the randomization (or an appropriate sequence generation)? | Yes |  | 100 |  |  |
|  | No |  | 0 |  |  |
|  | Can’t tell |  | 0 |  |  |
| QNR2. Is there a clear description of the allocation concealment (or blinding when applicable)? | Yes |  | 50 |  |  |
|  | No |  | 0 |  |  |
|  | Can’t tell |  | 50 |  |  |
| QNR3. Are there complete outcome data (80% or above)? | Yes |  | 50 |  |  |
|  | No |  | 50 |  |  |
|  | Can’t tell |  | 0 |  |  |
| QNR4. Is there low withdrawal/drop-out (below 20%)? | Yes |  | 50 |  |  |
|  | No |  | 0 |  |  |
|  | Can’t tell |  | 50 |  |  |
| QL1. Are the sources of qualitative data (archives, documents, informants, observations) relevant to address the research question (objective)? | Yes |  |  | 100 | 100 |
|  | No |  |  | 0 | 0 |
|  | Can’t tell |  |  | 0 | 0 |
| QL2. Is the process for analyzing qualitative data relevant to address the research question (objective)? | Yes |  |  | 100 | 100 |
|  | No |  |  | 0 | 0 |
|  | Can’t tell |  |  | 0 | 0 |
| QL3. Is appropriate consideration given to how findings relate to the context, e.g., the setting, in which the data were collected? | Yes |  |  | 100 | 100 |
|  | No |  |  | 0 | 0 |
|  | Can’t tell |  |  | 0 | 0 |
| QL4. Is appropriate consideration given to how findings relate to researchers’ influence, e.g., through their interactions with participants? | Yes |  |  | 45 | 100 |
|  | No |  |  | 0 | 0 |
|  | Can’t tell |  |  | 55 | 0 |
| MM1. Is the mixed methods research design relevant to address the qualitative and quantitative research questions (or objectives), or the qualitative and quantitative aspects of the mixed methods question (or objective)? | Yes |  |  |  | 100 |
|  | No |  |  |  | 0 |
|  | Can’t tell |  |  |  | 0 |
| MM2. Is the integration of qualitative and quantitative data (or results) relevant to address the research question (objective)? | Yes |  |  |  | 100 |
|  | No |  |  |  | 0 |
|  | Can’t tell |  |  |  | 0 |
| MM3. Is appropriate consideration given to the limitations associated with this integration, e.g. the divergence of qualitative and quantitative data? | Yes |  |  |  | 0 |
|  | No |  |  |  | 0 |
|  | Can’t tell |  |  |  | 100 |

QND: quantitative descriptive studies; QNR: quantitative randomized controlled trials; QL: qualitative studies; MM: mixed-method studies
