# Supplementary material for: Using Consolidated Framework for Implementation Research to investigate facilitators and barriers of implementing alcohol screening and brief intervention among primary care health professionals: a systematic review
Source: Implement Sci. 2021 Nov 20;16:99. doi: 10.1186/s13012-021-01170-8 (PMC8605518; doi:10.1186/s13012-021-01170-8)
Supplement: Supplementary file 3 — Additional file 3. [file 13012_2021_1170_MOESM3_ESM.docx]

Additional file 3 Studies reporting uptake rate of SBI.

| Study | Country | Ever performed alcohol screening or brief intervention (% of health professionals) | Performed alcohol screening or brief intervention regularly (% of health professionals) |
| --- | --- | --- | --- |
| Aalto (2006) | Finland | 59.4% | 9.4% |
| Aalto (2003) | Finland | 45% | N.A. |
| Costa (2019) | France | 94.1% | 31.7% |
| Farmer (2001) | UK | ≥80% | N.A. |
| Ferguson (2003) | USA | ≥60.0% | N.A. |
| Gordon (2011) | USA | ≥84.0% | 32.0% |
| Gurugama (2003) | Sri Lanka | N.A. | 15.0% |
| Holmqvist (2008) | Sweden | NA | 36.1% |
| Koopman (2008) | South Africa | 100% | 22.0% |
| Kraus (2017) | Germany | 84.2% | N.A. |
| Marcell (2002) | USA | 59.0% | N.A. |
| Rush (1994) | Canada | N.A. | 75% |
| Seppanen (2012) | Finland | 78.5% | 17.2% |
| Spandorfer (1999) | USA | ≥95.0% | 34.4% |
| Wilson (2011) | UK | 98.0% | 40.0% |
